# Children with HIV: A scoping review of auditory processing skills

**DOI:** 10.1371/journal.pone.0221573

**Published:** 2019-09-12

**Authors:** Gouwa Dawood, Daleen Klop, Elrietha Olivier, Haley Elliott, Mershen Pillay

**Affiliations:** 1 Division of Speech, Language and Hearing Therapy, Faculty of Medicine and Health Sciences, Stellenbosch University, Tygerberg, Cape Town, South Africa; 2 Discipline of Speech-Language Pathology, School of Health Sciences, University of KwaZulu-Natal, Westville, Durban, South Africa; Universidade Estadual de Ciencias da Saude de Alagoas, BRAZIL

## Abstract

**Introduction:**

Auditory processing disorders can negatively affect academic performance in children. They can result from a number of aetiologies, including the human immunodeficiency virus (HIV). Although studies in paediatrics are limited, research suggests that HIV-infected children display poorer auditory processing skills than uninfected children.

**Methods:**

The aims of this study were to scan the peer-reviewed literature on auditory processing skills in HIV-infected children, to describe how auditory processing was tested, how auditory processing skills were reported, and to identify gaps in current evidence. This systematic scoping review was conducted using a modified version of Arksey and O’Malley’s framework. Key words comprised ‘HIV’, ‘auditory processing’, ‘hearing’ and ‘child’. Electronic databases were searched for relevant articles published from 1 January 2000 to 30 April 2018, and reference lists of included studies were pearled. Two researchers reviewed the articles and extracted data on sample descriptors, auditory processing testing procedures, and auditory processing skills. A third author collated the results and resolved discrepancies. The American Speech-Language-Hearing Association description of auditory processing skills framed the analysis.

**Results:**

Five articles were included in this review (three from Brazil, one each from Mexico and Tanzania). Samples, and methods of testing were heterogeneous. Three studies reported on localization abilities, while gap detection thresholds, performance on dichotic tasks and speech discrimination scores were reported in one article each. No one study tested all areas of auditory processing skills and there was limited information about the auditory processing skills required for learning.

**Conclusion:**

This review highlighted the current sparse evidence-base for auditory processing in HIV-infected children. It identified the need to standardise testing procedures, measures of auditory processing skills, and sample selection.

## Introduction

Effective drug regimens have resulted in decreasing mortality rates from the human immunodeficiency virus (HIV) [1]. This has shifted attention to the impact of HIV on the developmental and educational outcomes of the approximately 1.8 million children, who are currently living with HIV [2]. HIV-infected children have been shown to perform poorer academically than their non-infected peers, with 40% of infected children aged between six and 12 years, being in a lower grade than is appropriate for their age [3]. It has therefore been suggested that HIV-infected children be recognised as a group with distinct educational needs [4]. However, in order to address their specific educational needs, research is required to describe the associated conditions affecting educational achievement.

Research has shown an association between hearing loss and HIV in the paediatric population with prevalence rates ranging from 6% to 84.4% [3,5–13]. Hearing loss in this population may be caused by the virus itself, opportunistic diseases and associated treatment regimens [14]. Hearing loss, which leads to reduced access to auditory information, significantly impacts a child’s ability to listen and learn in a mainstream classroom environment [15]. However, listening difficulties may not only be associated with hearing loss but more subtly, a child can have normal hearing but an impaired ability to process auditory information [16].

Auditory processing can be described as assigning meaning to what has been heard [17]. Discreet auditory processing skills form the foundation for listening [18]. These skills include: sound localization and lateralization, auditory discrimination, auditory temporal processing, auditory pattern processing, dichotic listening, auditory performance in competing acoustic signals, and auditory performance with degraded acoustic signals [19]. Whilst there is disagreement in the international literature about how or where auditory processing difficulties originate, for children with these deficits, the ability to function in a typical class environment is diminished [20].

Overall, research within HIV-infected populations has primarily reported on peripheral hearing loss [21], with few studies reporting on auditory processing. Although findings suggestive of central auditory deficits have been reported [5,8,22,23], these studies looked at the integrity of the auditory pathway rather than specific auditory processing skills. Matas et al [24], however, found that HIV-infected children were poorer at localizing sound than uninfected children. These findings have important implications for the paediatric HIV population, as disorders affecting auditory processing, in the absence of hearing loss, have been associated with poorer educational outcomes [25].

### Objective

The aim of this review was to systematically scan the published literature to identify papers of any research design that reported on auditory processing skills in the HIV-infected paediatric population.

## Methods

### Study design

A scoping review entails mapping key concepts and summarising available evidence of a particular research area [26,27]. A scoping review was the most appropriate design to address the objectives of this study, because so little is known about this area [28].

### Protocol registration

No protocol was registered for this scoping review as it was part of a bigger study that required the protocol to be registered by the Health Research Ethics Committee, Stellenbosch University.

### Reporting standard

This paper uses the PRISMA-ScR checklist [29] as a reporting standard. The review was conducted according to the framework proposed by Arksey and O’Malley [26], which was further developed by Levac, Colquhoun and O’Brien [27].

### Eligibility criteria

The search was conducted from 1 January 2000 until 30 April 2018. A PIO search framework was set, where P (patient) reflected children up to 18 years with HIV, I (intervention) was the assessment measure(s) for auditory processing skill(s), and O (outcome) was the auditory processing skill. Studies that included both adults and children were excluded. The inclusion and exclusion criteria are provided in Table 1.

**Table 1:** Inclusion and exclusion criteria.

Inclusion	Exclusion
Article reported on a specific auditory processing skill; namely sound localization and lateralization, auditory discrimination, auditory temporal processing, auditory pattern processing, dichotic listening, auditory performance in competing acoustic signals, and auditory performance with degraded acoustic signals [19]	Article did not refer to a specific auditory processing skill
Peer-reviewed journal articles	Non peer-reviewed journal articles, conference presentations and other sources of grey literature
Research/data driven articles only	Literature reviews and commentaries
Articles written in English	Articles not written in English
Study participants were HIV-infected children (18 years of age and under)	Study participants were HIV-infected adults (>18 years of age)

### Information sources and search

A comprehensive search strategy was designed in collaboration with a librarian from Stellenbosch University Library (Cape Town, South Africa). The databases searched included EBSCOhost research, Pubmed, Web of Science and Scopus. Within the EBSCOhost research database the following databases were accessed: Academic Search Premier, Africa Wide Information, CINAHL, Health Source: Nursing/Academic edition & MEDLINE.

These databases were searched using a combination of keywords including: HIV, auditory processing, hearing and child. The keywords or MeSH (Medical Subject Headings) terms were adapted according to the indexing of the database. While HIV is often associated with AIDS in the literature, preliminary testing of the search strategy identified that the inclusion in the search of the term ‘AIDS’ identified many irrelevant articles (e.g. where aids referred to assistive devices). This term was then removed from the search on the understanding that its absence in connection with HIV would not diminish the search parameters.

### Study selection

**Fig 1:**
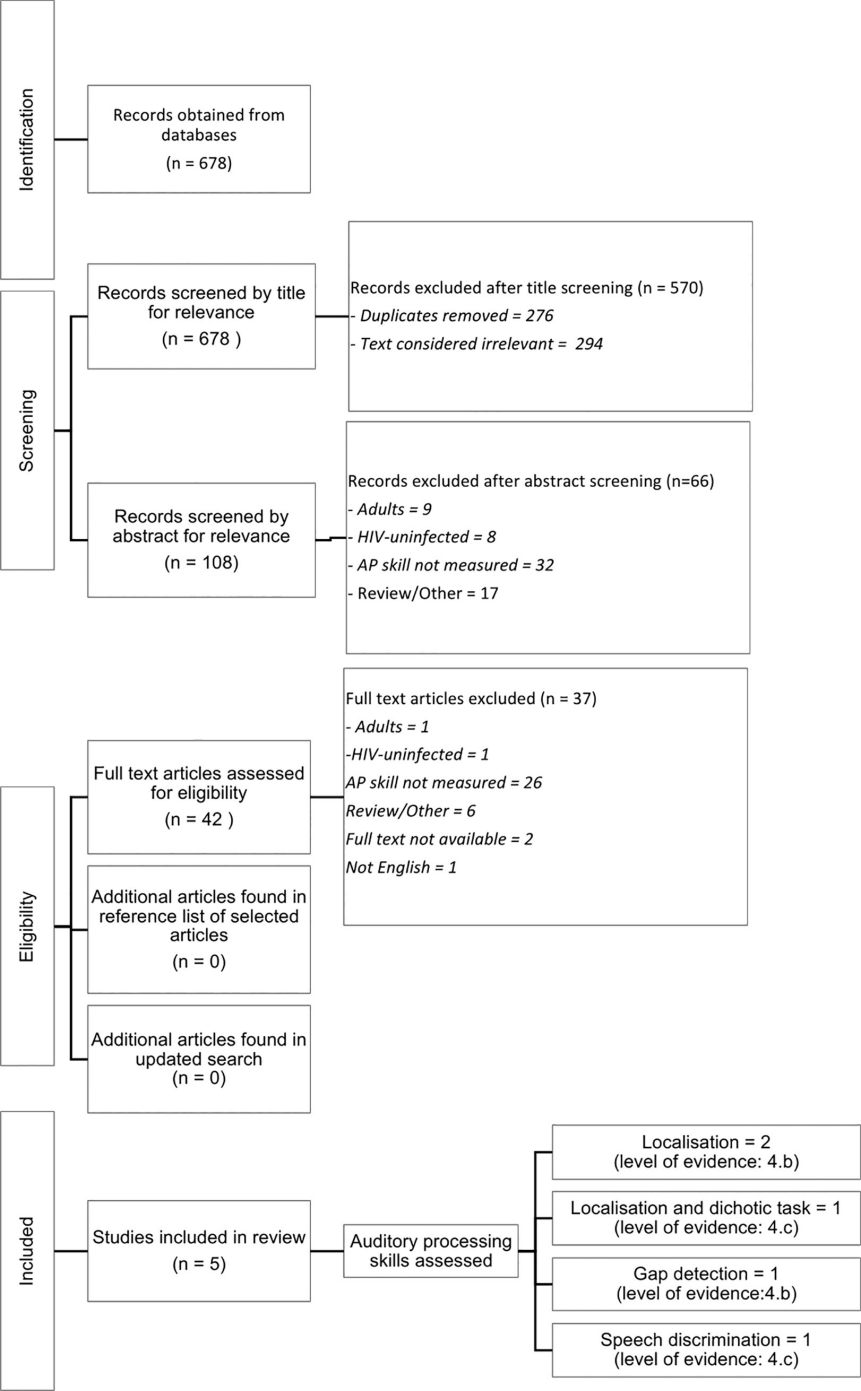
Flowchart depicting study selection process.

Studies identified by the search were first screened for duplicates, then for relevance. A three-step process was followed: the title was first checked for articles that were clearly not relevant for the purposes of the review (e.g. *Management of therapeutic hypothermia for neonatal hypoxic ischaemic encephalopathy in a tertiary centre in South Africa* was excluded, as it was not relevant to the focus of the review). Of the retained articles after this step, the abstracts were then read and further exclusions were made in line with the exclusion criteria (e.g. *Auditory Impairment in HIV-infected individuals in Tanzania* was excluded as it related to the adult population and did not report on a specific auditory processing skill). Articles were then read in full text, and retained if they focused on measurements of auditory processing skills in children who were HIV positive (Fig 1). Reference lists of the retained articles were then searched for additional studies that may not have been identified by the primary search. As a final step, an international expert on auditory processing was consulted on whether the auditory skills identified from each article, were correctly identified.

### Data collection process

A data extraction sheet (Table 2) was developed and used to extract data from included articles. The data collection sheet included: author, year, study design, level of evidence, study setting, description of the sample, assessment measures used in each study, auditory processing skill as described by the ASHA [19] and key findings. All stages of the review were conducted by two authors (EO & HE) who worked independently. Findings were then compared, and any discrepancies were discussed and resolved by EO and HE. A third author (GD), who also collated the results, was consulted when a discrepancy could not be resolved.

**Table 2:** Key characteristics of included articles as these relate to auditory processing skills.

Author(s)	Title	Study design (Level of evidence)	Study setting	Participants (Patient)	Assessment measure (Intervention)	Auditory processing skill (ASHA, 2005) (Outcome)	Key findings
Matas, Sansone, Iorio, & Succi (2000)	Audiological evaluation in children born to HIV positive mothers	Cross sectional Case-control (Level 4.b)	São Paulo, Brazil (urban)	143 children aged 1 month to 30 months (HIV+ = 18, HEP = 34, HEU = 91)	BOA (size of response, timing of response, attention to sound, lateralization, localization in vertical plane, cochlea-palpebral reflex	Binaural interaction (lateralization/localization)	Central auditory impairment observed more often in HIV+ group than in two control groups. In HIV+ group, findings suggestive of central auditory disorder observed more frequently than findings indicating middle ear involvement.
Matas, Iori, Succi & CecÍlia (2008)	Auditory disorders and acquisition of the ability to localize sound in children born to HIV-positive mothers	Cross sectional Case-control (Level 4.b)	São Paulo, Brazil (urban)	143 children aged 1 month to 30 months (HIV+ = 18, HEP = 34, HEU = 91)	BOA (size of response, timing of response, attention to sound, lateralization, localization in vertical plane, cochlea-palpebral reflex)	Binaural interaction (lateralization/localization)	Significant difference between HIV+ group and two control groups with regards to acquisition of ability to localise sound.
Palacios, Montalvo, Fraire, Leon, Alvarez & Solorzano (2008)	Audiologic and vestibular findings in a sample of Human Immunodeficiency Virus type-1- infected Mexican children under highly active antiretroviral therapy	Cross sectional Case-series (Level 4.c)	Mexico City, Mexico (urban)	23* HIV+ children aged 5 months to 16 years No control group	Speech discrimination (9 participants)	Auditory discrimination	Abnormalities in speech discrimination observed in 4 children: 2 suggesting conductive involvement, 1 cochlear involvement and 1 central involvement observed in 1 child
Maro et al. (2016)	Auditory impairments in HIV-infected children	Cross sectional Case-control (Level 4.b)	Dar es Salaam, Tanzania (urban)	244* children aged younger than 18 years (HIV+ = 131, HIV- = 113)	Gap detection (HIV+ = 48, HIV- = 19) sample size as reflected in Results section and not in Abstract	Auditory temporal processing and patterning	No significant difference in gap detection thresholds and ABR latencies between the HIV infected and control children. ABR latencies for HIV- group reflected in text 0.1msec longer than latency reflected in Results section.
Romero, Alfaya, Gonçales, Frizzo & Isaac (2017)	Auditory alterations in children infected by Human Immunodeficiency Virus verified through auditory processing test	Cross sectional Case series (Level 4.c)	Sao Paula, Brazil (urban)	15 children aged 8 to 9 years No control group	SSW, SAPT (sound localization in 5 directions, memory for verbal sounds, memory for nonverbal sounds)	Binaural integration (dichotic speech), binaural interaction (localisation)	Auditory changes, related to auditory processing, observed. Difficulties observed related to deficits in attention, memory and auditory figure ground skills. 8-year olds performed poorer than 9-year olds suggesting a maturational effect.

*Total number of children who underwent audiological assessment, not necessarily auditory processing assessment

HIV+ = children infected with HIV; HIV- = children who are HIV negative; HEU = children who have been exposed to the virus but are uninfected; HEP = children who have been exposed to the virus and are positive but their status has not been confirmed due to their age.

BOA = behavioural observation audiometry, SSW = staggered spondaic words, SAPT = simplified auditory processing test

### Hierarchy of evidence

Study design was classified according to the Joanna Briggs Institute (JBI) levels of evidence [30].

### Assessment of risk of bias

Methodological quality of included studies was not evaluated due to the scoping nature of the review [29].

### Synthesis of results

Data was summarized for currency, country of origin, method of assessing auditory processing skills and key outcome measures using tables, figures, numerical analysis and narrative synthesis, as recommended by Arksey and O’Malley [26].

### Additional analyses

By examining the scope of research described in each article, this enabled the authors to identify gaps in literature and areas for further research.

## Results

### Study selection

Fig 1 outlines the study selection process. A total of 678 articles were found in the selected databases. After titles were screened for relevance and duplicates were removed, 108 articles remained. Abstracts of these articles were then screened, according to the inclusion and exclusion criteria (Table 1), and resulted in 42 articles being included for full text review. Following full text review, 37 articles were excluded as they did not meet inclusion and exclusion criteria (Table 1). The remaining five articles [5,23,24,31,32] were retained for this review. Table 2 summarises key characteristics of each article. No additional articles were found during the search of relevant reference lists.

### Study characteristics

The studies were all cross-sectional designs, whose subjects ranged in age from 1 month to 16 years. Although the study sample sizes ranged from 15 to 244 children, auditory processing skills were not measured in all the subjects. Three of the studies included control groups [23,24,32], while two studies reported case series [5,31]. The studies were all conducted in urban settings, in developing countries, with three of the studies based in Brazil (South America) and the other two studies based respectively in Tanzania (Africa) and Mexico (North America). Table 2 provides a summary of the included studies, and Table 3 summarises the reference population and the sample.

**Table 3:** Reference population and sampling approach.

Study	Nationality	Reference population	Source	HIV diagnosis	Age
Palacios et al 2008	Mexico	HIVP children < 17 years	AIDS outpatient clinic	all infected	5mths—17 years
Matas et al 2000	Brazil	Children born to HIV-infected mothers	Department of Pediatrics	HIV children (I), serum-reverted (SR) and exposed to HIV (I).	1mth-2.5yrs
Romero et al 2017	Brazil	Children with HIV	not stated	all infected	8 or 9 years
Matas et al 2008	Brazil	Children born to HIV-infected mothers	Department of Pediatrics	HIV children (I), serum-reverted (SR) and exposed to HIV (I).	1mth-2.5yrs
Maro et al 2016	Tanzania	HIVP children < 18 years	Pediatric Program at Infectious Disease Center	HIV+ children and HIVN family members	0.8 yrs-to 18 yrs

### Synthesis of the results

#### Sampling issues

The five papers reported on four datasets, with Matas et al (2000, 2008) reporting on different aspects of the same sample. The study samples differed in selection criteria, age and health status. Although four papers reported on the sampling location, information on how children were selected for study participation was notably absent in all studies. Overall, the included papers report on 185 HIVP children (cases), of a total of 425 children. All cases were on ART. Within the 240 non-HIVP (control) children were 78 serum-reverted children and 30 HIV exposed children, who were treated separately in subgroup analysis (Matas et al 2000, 2008). Maro et al (2016) recruited 113 HIVN (control) children who were family members of cases (Maro et al 2016). Whilst four studies excluded children with comorbid medical conditions, Maro et al (2016) included 31 HIVP and 3 HIVN children with TB histories. The overall sample age ranged from one month to 18 years, however it was not possible from the available information to estimate an average sample age. On the available data, it might be presumed that the majority of children were younger than 10 years. Palacios et al (2008) reported on 23 Mexican cases (average age 4.5 years); Matas et al (2000, 2008) reported on a dataset of 143 children born to HIVP mothers (all children being younger than 2.5 years); Maro et al (2016) reported on 244 children (cases and controls) of average age 10.1 years, and Romero et al (2017) reported on 15 cases aged 8 or 9 years. Sample heterogeneity potentially underpins the lack of consistency in the findings of this review.

#### Auditory processing testing procedures

The studies described different test procedures, namely Behavioural Observation Audiometry (size of response, timing of response, attention to sound, lateralization, localization in vertical plane, cochlea-palpebral reflex) (Matas et al 2000, 2008); speech discrimination (Palacios et al 2017); gap detection thresholds (Maro et al 2016); and Staggered Spondaic Words, Simplified Auditory Processing Test (sound localization in 5 directions, memory for verbal sounds, memory for nonverbal sounds) (Romero et al 2017).

#### Auditory processing skills

Moreover, the test procedures described in the studies assessed different auditory processing skills, Four clusters of skills, described below, were identified during data extraction: (1) binaural interaction (Matas et al 2000, 2008, Romaro 2017); (2) auditory discrimination (Palacios et al 2017); (3) auditory temporal processing (Maro et al 2016) and (4) binaural integration (Romero et al, 2017).

- **Binaural interaction:** Localisation, as a measure of binaural interaction, was assessed in three studies [24,31,32]. A higher occurrence of abnormal findings in the HIV-infected group, compared to the two uninfected groups, was reported by Matas et al [24,32] while Romero et al [31] reported that localisation difficulties occurred in less than 30% of their participants.
- **Auditory discrimination:** The only study that assessed auditory discrimination skills [5], reported that abnormal findings were observed in four children in a sample of nine. The origin of the deficits were attributed to conductive involvement (n = 2), cochlear involvement (n = 1) and central involvement (n = 1).
- **Auditory temporal processing:** Maro et al [23] reported on gap detection thresholds as the outcome measure for auditory temporal processing. The authors reported that the mean and median gap detection thresholds, between children infected with HIV (n = 48) and uninfected children (n = 19) were not significantly different.
- **Binaural integration:** One study assessed binaural integration by using the Staggered Spondiac Word Test (SSW) [31]. Although 87% (13 of the 15 participants) of children presented with difficulties, these difficulties were attributed to problems in the areas of attention, memory and auditory figure-ground skills rather than only problems with binaural integration.

## Discussion

This is the first scoping review that we know of, that describes the volume and nature of research relating to auditory processing skills in HIV-infected children. We believe that our scoping review search strategy was sufficiently comprehensive that we identified all available evidence in this area. The strength of the scoping review was enhanced by having two independent researchers undertake the screening and extraction steps. Moreover, the scoping nature of the review enabled the identification of all available literature in this area without the restraints of seeking specific research designs or methodological quality. It thus provided a comprehensive overview of the literature currently available on the topic.

From the limited available literature, there is consistent evidence of deficits in the auditory processing skills that were assessed in HIV-infected children [5,23,24,31,32]. The review highlights that the published research is limited in numbers of studies (five), study designs (cross-sectional), geographical settings (urban, developing countries).

A possible reason for the limited research on auditory processing in the HIV-infected paediatric population, is the lack of consensus between researchers on definitions and/or descriptions of auditory processing [33]. Various definitions have been postulated with the definition by the American Speech-Language-Hearing Association [19] being the most widely cited. According to this definition, (Central) Auditory Processing is *“the perceptual processing of auditory information in the CNS and the neurobiologic activity that underlies that processing and gives rise to electrophysiologic auditory potentials”*. More recently, the British Society of Audiology [34] has described auditory perception as being *“the awareness of acoustic stimuli*, *forming the basis for subsequent action*” and *“results from both sensory activation (via the ear) and neural processing that integrates this ‘bottom up’ information with activity in other brain systems (e*.*g*. *vision*, *attention*, *memory)”*.

The literature identified in this review identified limitations in the scope of assessment measures for auditory processing skills. The use of assessment measures as a reflection of a specific skill, is complex. A gold standard for the assessment of auditory processing disorders does not exist, although various assessment guidelines recommend that a test battery approach be used and that an auditory processing deficit cannot be diagnosed on the basis of one test, measuring a single auditory processing skill [19,35–38]. The American Speech-Language-Hearing Association [19] lists various categories of central auditory tests that may be considered for the assessment of central auditory processing including: auditory discrimination tests, auditory temporal processing, dichotic speech tests, monaural low-redundancy speech test, binaural interaction tests, electroacoustic measures and electrophysiologic measures. Despite the variety of assessment measures available, four of the five studies included in the review, only reported on measures that were easily quantifiable and not language-dependant, such as auditory discrimination, localization and gap detection thresholds.

Auditory temporal processing do not appear to be affected by HIV [23]. Maro et al. [23] did not report significant differences in gap detection thresholds between HIV-infected and uninfected children. Furthermore, similar performances were reported for HIV-infected children regardless of the type of ART being used or the time delay before initiation of treatment [23]. A possible reason for the lack of statistically significant results was the small sample size [39] as very few children could complete the task due to the complexity of the instructions. Despite similar results being reported for adults when HIV-infected and uninfected individuals were compared [40], within the group of HIV-infected adults, persons using antiretroviral therapy (ART) performed significantly poorer than persons not using medication [40]. Based on the findings of this adult study, a possible explanation for deficits in gap detection is the use of ART, rather than the virus itself. However, the two groups that were compared in the adult study also differed in term of mean age and history of tuberculosis [40] and both these factors have been associated with auditory dysfunction [41,42].

Research suggests that HIV may affect auditory discrimination. The only study that used this skill as an outcome measure, reported that 44% of the sample presented with deficits [5]. However, this study was a case series with a small sample size (nine participants). Although the authors differentiated between conductive, cochlear and central impairment; the inclusion of children with hearing loss may have increased the prevalence of abnormal findings as hearing loss has been associated with impaired speech perception abilities [43].

Binaural interaction skills appear to be impaired in HIV-infected children. This can be seen in deficits in localizing abilities [24,31,32]. However, the extent to which these abilities are affected is not known as one of the studies was a case series [31] which provides limited information about causality and the pattern of the auditory processing deficits [39]. The other studies were case-control studies [24,32] that did not report on the statistical significance of the observed differences. A factor that may have contributed to the prevalence of impaired localizing skills is age, as two of the studies [24,32] assessed babies and infants (interrogating the same sample of children born to HIVP mothers). Muir et al. [44] reported that the development of localizing skills followed a u-shaped trajectory. According to this trajectory, a neonate’s response to sound decreases between the ages of one and three months with a greater, more accurate response being seen from four to five months of age [44].

Binaural integration is reported to be abnormal in HIV-infected children. The findings reported by the one study that assessed performance on a dichotic speech test [31] are consistent with the findings reported for a study conducted in HIV-infected adults [45]. However, when considering the well-documented neurocognitive effects of HIV [46,47], it is difficult to comment on whether it is a pure binaural integration deficit as this skill is so intimately entwined with executive functions, in particular attention and memory [48].

The biologic sequelae of HIV have been well-documented in children, and the studies included in this review provide insights into a few auditory processing skills that are affected in HIV-infected children. These studies use methodologies that answer questions related to the organic nature of HIV and provide information on the deficits that may be associated with HIV. However, when one considers the chronic nature of the virus, these methodologies may need to be supplemented with measures to answer questions related to functional outcomes of HIV. In order to provide appropriate educational services, identifying deficits that may affect learning is not enough, as understanding how these deficits affect the way the individual functions in their learning environment, is also needed.

### Limitations

The review findings highlight the scarcity of research in this area. We were concerned that the inclusion of only English language studies may have introduced bias toward countries from Europe and North America, however, all the articles were from countries whose official languages were not English. The exclusion of conference abstracts and other grey literature may have also introduced publication bias; however, this literature may not have provided the detail that we required in order to describe research in the area.

## Conclusion

This systematic scoping review identified only five studies on auditory processing skills in HIV-infected children and highlights the paucity of research in the area. This evidence-base is inconclusive regarding the association between HIV and auditory processing difficulties as the studies did not necessarily assess the same auditory processing skill and findings were, therefore not comparable. In order to better understand the impact of the virus on learning and to justify the inclusion of auditory processing assessments in the basic health and educational services offered to children infected with HIV, further research of all auditory processing skills is needed. Carefully designed case control studies looking at the time-sequence and relationship between HIV, auditory processing and learning in children should be considered. As the organic consequences of HIV have frequently been demonstrated, it is imperative that future studies include functional outcomes so as to develop a more comprehensive picture of the auditory abilities of this population. Furthermore, research needs to address issues of sampling, sample size calculation and heterogeneity of the population, as well as exerting better controls for maturational aspects of auditory processing, by carefully considering the age of both participants and controls.

## Supporting information

S1 Checklist PRISMA-ScR checklist. (DOCX)

S1 Dataset S1 Dataset. (DOCX)
