# Supplementary material for: Children with HIV: A scoping review of auditory processing skills
Source: PLoS One. 2019 Sep 12;14(9):e0221573. doi: 10.1371/journal.pone.0221573 (PMC6742466; doi:10.1371/journal.pone.0221573)
Supplement: S1 Dataset — (DOCX) [file pone.0221573.s002.docx]

| **Author(s)** | **Title** | **Study design (Level of evidence)** | **Study setting** | **Participants (Patient)** | **Assessment measure (Intervention)** | **Auditory processing skill (ASHA, 2005) (Outcome)** | **Key findings** |
| --- | --- | --- | --- | --- | --- | --- | --- |
| Matas, Sansone, Iorio, & Succi (2000) | Audiological evaluation in children born to HIV positive mothers | Cross sectional  Case-control  (Level 4.b) | São Paulo, Brazil (urban) | 143 children aged 1 month to 30 months (HIV+ = 18, HEP = 34, HEU = 91) | BOA (size of response, timing of response, attention to sound, lateralization, localization in vertical plane, cochlea-palpebral reflex | Binaural interaction (lateralization/localization) | Central auditory impairment observed more often in HIV+ group than in two control groups. In HIV+ group, findings suggestive of central auditory disorder observed more frequently than findings indicating middle ear involvement. |
| Matas, Iori, Succi & CecÍlia (2008) | Auditory disorders and acquisition of the ability to localize sound in children born to HIV-positive mothers | Cross sectional  Case-control  (Level 4.b) | São Paulo, Brazil (urban) | 143 children aged 1 month to 30 months (HIV+ = 18, HEP = 34, HEU = 91) | BOA (size of response, timing of response, attention to sound, lateralization, localization in vertical plane, cochlea-palpebral reflex) | Binaural interaction (lateralization/localization) | Significant difference between HIV+ group and two control groups with regards to acquisition of ability to localise sound. |
| Palacios, Montalvo, Fraire, Leon, Alvarez & Solorzano (2008) | Audiologic and vestibular findings in a sample of Human Immunodeficiency Virus type-1- infected Mexican children under highly active antiretroviral therapy | Cross sectional  Case-series  (Level 4.c) | Mexico City, Mexico (urban) | 23* HIV+ children aged 5 months to 16 years  No control group | Speech discrimination (9 participants) | Auditory discrimination | Abnormalities in speech discrimination observed in 4 children: 2 suggesting conductive involvement, 1 cochlear involvement and 1 central involvement observed in 1 child |
| Maro et al. (2016) | Auditory impairments in HIV-infected children | Cross sectional  Case-control  (Level 4.b) | Dar es Salaam, Tanzania (urban) | 244* children aged younger than 18 years (HIV+ = 131, HIV- = 113) | Gap detection (HIV+ = 48, HIV- = 19) sample size as reflected in Results section and not in Abstract | Auditory temporal processing and patterning | No significant difference in gap detection thresholds and ABR latencies between the HIV infected and control children. ABR latencies for HIV- group reflected in text 0.1msec longer than latency reflected in Results section. |
| Romero, Alfaya, Gonçales, Frizzo & Isaac (2017) | Auditory alterations in children infected by Human Immunodeficiency Virus verified through auditory processing test | Cross sectional  Case series  (Level 4.c) | Sao Paula, Brazil (urban) | 15 children aged 8 to 9 years  No control group | SSW, SAPT (sound localization in 5 directions, memory for verbal sounds, memory for nonverbal sounds) | Binaural integration (dichotic speech), binaural interaction (localisation) | Auditory changes, related to auditory processing, observed. Difficulties observed related to deficits in attention, memory and auditory figure ground skills. 8-year olds performed poorer than 9-year olds suggesting a maturational effect. |

| **Study** | **Nationality** | **Reference population** | **Source** | **HIV diagnosis** | **Age** |
| --- | --- | --- | --- | --- | --- |
| Palacios et al 2008 | Mexico | HIVP children < 17 years | AIDS outpatient clinic | all infected | 5mths - 17 years |
| Matas et al 2000 | Brazil | Children born to HIV-infected mothers | Department of Pediatrics | HIV children (I), serum-reverted (SR) and exposed to HIV (I). | 1mth-2.5yrs |
| Romero et al 2017 | Brazil | Children with HIV | not stated | all infected | 8 or 9 years |
| Matas et al 2008 | Brazil | Children born to HIV-infected mothers | Department of Pediatrics | HIV children (I), serum-reverted (SR) and exposed to HIV (I). | 1mth-2.5yrs |
| Maro et al 2016 | Tanzania | HIVP children < 18 years | Pediatric Program at Infectious Disease Center | HIV+ children and HIVN family members | 0.8 yrs-to 18 yrs |

Data sources:

1. Matas CG, Sansone AP, Iorio MCM, Succi RCM. Audiological evaluation in children born to HIV positive mothers. Braz J Otorhinolaryngol. 2000;66(4):317–24.

2. Matas CG, Iorio MMCM, Succi RCDM, Cecília M. Auditory Disorders and Acquisition of the Ability to Localize Sound in Children Born to HIV-Positive Mothers. Brazilian J Infect Dis. 2008;12(1):10–4.

3. Palacios GC, Montalvo MS, Fraire MI, Leon E, Alvarez MT, Solorzano F. Audiologic and vestibular findings in a sample of Human Immunodeficiency Virus type-1-infected Mexican children under Highly Active Antiretroviral Therapy. Int J Pediatr Otorhinolaryngol. 2008 Nov;72(11):1671–81.

4. Maro II, Fellows AM, Clavier OH, Gui J, Rieke CC, Wilbur JC, et al. Auditory Impairments in HIV-Infected Children. Ear Hear. 2016;37(4):443–51.

5. Romero A, Alfaya L, Gonçales A, Frizzo A, Isaac M. Auditory Alterations in Children Infected by Human Immunodeficiency Virus Verified Through Auditory Processing Test. Int Arch Otorhinolaryngol. 2016 Mar 14;21(01):86–91.
